# Supplementary material for: Surgical Interventions for Late Aortic Valve Regurgitation Associated with Continuous Flow-Left Ventricular Assist Device Therapy: Experience Gained and Lessons Learned
Source: Life (Basel). 2022 Dec 29;13(1):94. doi: 10.3390/life13010094 (PMC9867390; doi:10.3390/life13010094)
Supplement: Supplementary file 1 [file life-13-00094-s001.zip › life-2104254-supplementary.pdf]

Table S1. Summary of surgical intervention, intraoperative findings, and outcomes.

Patient No.	Procedure	Pathology of aortic valve	Prolapse	Degenerative change	Dilatation of annulus	Unknown	AR grade at 1 month after surgery	Outcomes
1	AVR, TAP		No	Yes	No	No	No	Dead
2	AVR		No	Yes	No	No	No	HTx
3	AVR		No	Yes	No	No	No	HTx
4	AVR		Yes	No	No	No	No	Alive
5	AVR		No	Yes	No	No	No	Alive
6	AVR		No	No	Yes	No	No	Alive
7	AVP+TVR		No	No	No	Yes	No	HTx
8	AVP+TVR		Yes	No	No	No	No	HTx
9	AVP		No	Yes	No	No	Severe	HTx
10	AVP		No	No	No	Yes	No	HTx

AVR, aortic valve regurgitation; TAP, tricuspid annuloplasty; AVP, aortic valve repair; TVR, tricuspid valve replacement; AR, aortic regurgitation; HTx, heart transplantation.
